# Perspectives in immunotherapy: meeting report from the immunotherapy bridge (December 2nd–3rd, 2020, Italy)

**DOI:** 10.1186/s12967-021-02895-2

**Published:** 2021-06-02

**Authors:** Paolo A. Ascierto, Carlo Bifulco, Fortunato Ciardiello, Sandra Demaria, Leisha A. Emens, Robert Ferris, Silvia C. Formenti, Jerome Galon, Samir N. Khleif, Tomas Kirchhoff, Jennifer McQuade, Kunle Odunsi, Akash Patnaik, Chrystal M. Paulos, Janis M. Taube, John Timmerman, Bernard A. Fox, Patrick Hwu, Igor Puzanov

**Affiliations:** 1 Department of Melanoma, Cancer Immunotherapy and Innovative Therapy, Istituto Nazionale Tumori IRCCS “Fondazione G. Pascale”, Naples, Italy; 2 Providence Cancer Center, Earle A. Chiles Research Institute, Portland, OR USA; 3 Medical Oncology and Hematology Division, University “Luigi Vanvitelli”, Naples, Italy; 4 Department of Radiation Oncology, Weill Cornell Medical College, New York, NY USA; 5 Sandra and Edward Meyer Cancer Center, New York, NY USA; 6 Department of Pathology and Laboratory Medicine, Weill Cornell Medical College, New York, NY USA; 7 Magee Women’s Hospital, Pittsburgh, PA USA; 8 UPMC Hillman Cancer Center, Pittsburgh, PA USA; 9 Weill Cornell Medicine and Meyer Cancer Center, New York, NY USA; 10 INSERM, Laboratory of Integrative Cancer Immunology, Paris, France; 11 Equipe Labellisée Ligue Contre le Cancer, Paris, France; 12 Centre de Recherche des Cordeliers, Sorbonne Université, Université de Paris, Paris, France; 13 The Loop Immuno Oncology Laboratory, Georgetown University Medical School, Washington, DC USA; 14 Perlmutter Cancer Center, New York, NY USA; 15 Melanoma Medical Oncology, MD Anderson Cancer Center, Houston, TX USA; 16 University of Chicago Medicine Comprehensive Cancer Center, Chicago, IL USA; 17 Department of Obstetrics and Gynecology, University of Chicago, Chicago, IL USA; 18 Section of Hematology/Oncology, Department of Medicine, The University of Chicago, Chicago, IL USA; 19 Winship Cancer Institute at Emory University, Atlanta, GA USA; 20 Department of Dermatology, Johns Hopkins University SOM, Baltimore, MD USA; 21 David Geffen School of Medicine, UCLA Medical Center, Los Angeles, CA USA; 22 Earle A. Chiles Research Institute, Robert W. Franz Cancer Research Center, Providence Cancer Institute, Portland, OR USA; 23 Moffitt Cancer Center, Tampa, FL USA; 24 Department of Medicine, Roswell Park Comprehensive Cancer Center, Buffalo, NY USA

**Keywords:** Immunotherapy, Checkpoint inhibitors, Combination therapy, Biomarkers, Tumor microenvironment, Vaccine

## Abstract

Improved understanding of tumor immunology has enabled the development of therapies that harness the immune system and prevent immune escape. Numerous clinical trials and real-world experience has provided evidence of the potential for long-term survival with immunotherapy in various types of malignancy. Recurring observations with immuno-oncology agents include their potential for clinical application across a broad patient population with different tumor types, conventional and unconventional response patterns, durable responses, and immune-related adverse events. Despite the substantial achievements to date, a significant proportion of patients still fail to benefit from current immunotherapy options, and ongoing research is focused on transforming non-responders to responders through the development of novel treatments, new strategies to combination therapy, adjuvant and neoadjuvant approaches, and the identification of biomarkers of response. These topics were the focus of the virtual Immunotherapy Bridge (December 2nd–3rd, 2020), organized by the Fondazione Melanoma Onlus, Naples, Italy, in collaboration with the Society for Immunotherapy of Cancer and are summarised in this report.

## Introduction

Over recent years, a substantial research effort has improved our understanding of tumor immunology and enabled the development of novel treatments that harness the immune system and prevent immune escape. Through numerous clinical trials and real-world experience, evidence of the potential for long-term survival with immunotherapy agents has accumulated in various types of malignancy. These studies have highlighted several recurring observations with immuno-oncology agents, including their potential for clinical application across a broad patient population across tumor types, both conventional and unconventional response patterns, durable responses, and immune-related adverse events. However, a significant proportion of patients are still failing to benefit from current immunotherapy options, and ongoing preclinical and clinical research is focused on transforming non-responders to responders, through the development of novel treatments, new approaches to combination therapy, adjuvant and neoadjuvant cancer immunotherapy, and the identification of biomarkers of response.

Biomarkers, drivers of immune response and trends in immunotherapy were the focus of the virtual Immunotherapy Bridge (December 2nd–3rd, 2020), organized by the Fondazione Melanoma Onlus, Naples, Italy, in collaboration with the Society for Immunotherapy of Cancer.

## SITC session—Biomarkers

### Multiplex immunofluorescence assay development: current status and future directions

Technologies are now available that allow for the simultaneous targeting of multiple proteins in formalin-fixed paraffin-embedded tissue samples, commonly referred to as multiplex immunohistochemistry (IHC) or immunofluorescence (IF). These approaches can distinguish between different cell types expressing the same protein and can characterize the density and spatial distribution of specific cells within the tumor microenvironment (TME). IF also has the benefit of being able to characterize a large dynamic range of expression on a cell-by-cell basis.

Consideration needs to be given to assay validation, especially with the increasing ‘plex’ of assays. Multiplex IF panel development is the consolidation of multiple monoplex IF protocols into a single protocol. Robust in situ assessment of intensity of biomarker expression, e.g., programmed death-1 (PD-1) and programmed death ligand-1 (PD-L1), in a reproducible manner is also desirable.

Best practice guidelines for multiplex IF assays have been developed [1]. One key standard is that the multiplex assay should be equivalent to the monoplex IF/IHC for each individual marker. Multiplex IF assays may show lower levels of marker detection than their individual assay components due to steric hinderance between the multiple markers, signal interference between the fluors, or different reagent properties. For example, we found in the development of a multiplex IF assay that the IF was less sensitive than chromogenic IHC for key markers such as PD-L1. Additional amplification using an alternative horseradish peroxidase (HRP) polymer maximized sensitivity of the IF and resulted in comparable sensitivity to chromogenic IHC. Final panel validation showed that the combined multiplex IF panel was similar to the monoplex IF for each marker. Such optimized assays have been shown to be reproducible in multi-institutional studies [2]. For example, a six-plex multiplex IF assay was shown to have high inter-site concordance for percent PD-L1 co-expression within different cell types. Proximity of different cell types was also shown to be reproducible with high inter-center concordance. Additional study is underway to assess whether higher-‘plex’ assays are comparable to their single-plex components and whether they are reproducible across multiple laboratories.

Further gains in multiplex IF accuracy and reproducibility can be achieved through considered cell segmentation and controlling for potential batch-to-batch variations. Cell segmentation is the process that image analysis algorithms use to identify the cell compartments of the membrane, cytoplasm, and nucleus. In immuno-oncology, where immune cells and tumor cells are often in close proximity, errors in cells segmentation often result in the membrane expression of an immunoactive marker being mistakenly attributed to a neighboring cell. Additionally, cell size is often a key component in the image analysis algorithms, and many algorithms struggle when there is a wide variation in cell sizes. We found that when we were trying to identify larger cells (tumor cells and macrophages) at the same time as smaller cells (lymphocytes), the accurate segmentation of lymphocytes led to over-segmentation (and thus over-counting) of the tumor cells and macrophages. We found that this could be corrected if the cells bearing each marker were segmented individually, rather than attempting to simultaneously segment cells displaying all markers [3]. Another important consideration is batch-to-batch staining variation, in particular when assessing intensity of expression. This can be controlled for by including a tissue microarray of control tissue with each batch, facilitating correction of marker expression intensity across batches; for example, normalizing to tissue controls can reduce the coefficient of variation in PD-1/PD-L1 expression intensity between batches from 10–15% to ~ 5%. Careful optimization of multiplex staining, cell segmentation, and correction for batch-to-batch variation allows for more accurate and robust assessments of marker intensity in situ and associated immuno-oncology biomarker development.

### Integrating multiomics in the practice of diagnostic pathology

Deep insights into the biology of cancer, combined with rapid advances in our understanding of the molecular biomarker landscape, are transforming the practice of oncology and are enabling the growth of personalized treatment indications, as illustrated by the ever-growing list of Food and Drug Administration (FDA) approved targeted therapies and in immuno-oncology by the recent pan-cancer approval of pembrolizumab in patients with a tumor mutational burden (TMB) ≥ 10 mut/Mb. Despite this progress, we still struggle to consistently translate this knowledge into clinical outcomes, as many patients often fail to receive appropriate therapies, with evidence coming from the non-small cell lung cancer (NSCLC) experience suggesting that less the half of eligible patients for biomarker driven personalized medicine ever receive a targeted therapy.

Multiple factors underly these disparities in clinical delivery. Among them, a significant role is played by the persistent failure to tightly integrate comprehensive genomic profiling into routine clinical diagnostic pathology workflows, and by the frequent persistence in clinical practice, of a narrow single or small panel gene reflex testing approach, where diagnostic targets are tested sequentially in a step-wise fashion, a process which consumes precious time and often also precious tissue, and is neither sustainable nor cost-efficient, and often does not enable the delivery of the correct treatment in a timely fashion.

This is despite the continuous progress in next generation sequencing technologies, nowadays enabling the comprehensive assessment of TMB, microsatellite instability (MSI) and somatic variants in over 500 cancer-related genes in a single workflow of more than 30,000 patients/year on a single high throughput sequencing platform. At the Providence Cancer Institute (Portland, OR, USA) comprehensive genomic profiling is integrated as a standard of care in routine diagnostic practices and executed automatically as early as possible (i.e., at the time of an initial tumor diagnosis by a pathologist) under an institutional review board-approved clinical improvement protocol. The implementation of such a program has resulted in a significant increase in the number of patients with detected actionable markers, leading to an increased enrolment in targeted therapy driven clinical trials and to an increased number of patients qualifying for immunotherapies because of a high TMB or MSI status. Importantly, beyond treatment selection, the tight coupling of genomics and pathology can benefit diagnostic workflows by informing also tumor classification, staging and diagnosis.

**Fig. 1:**
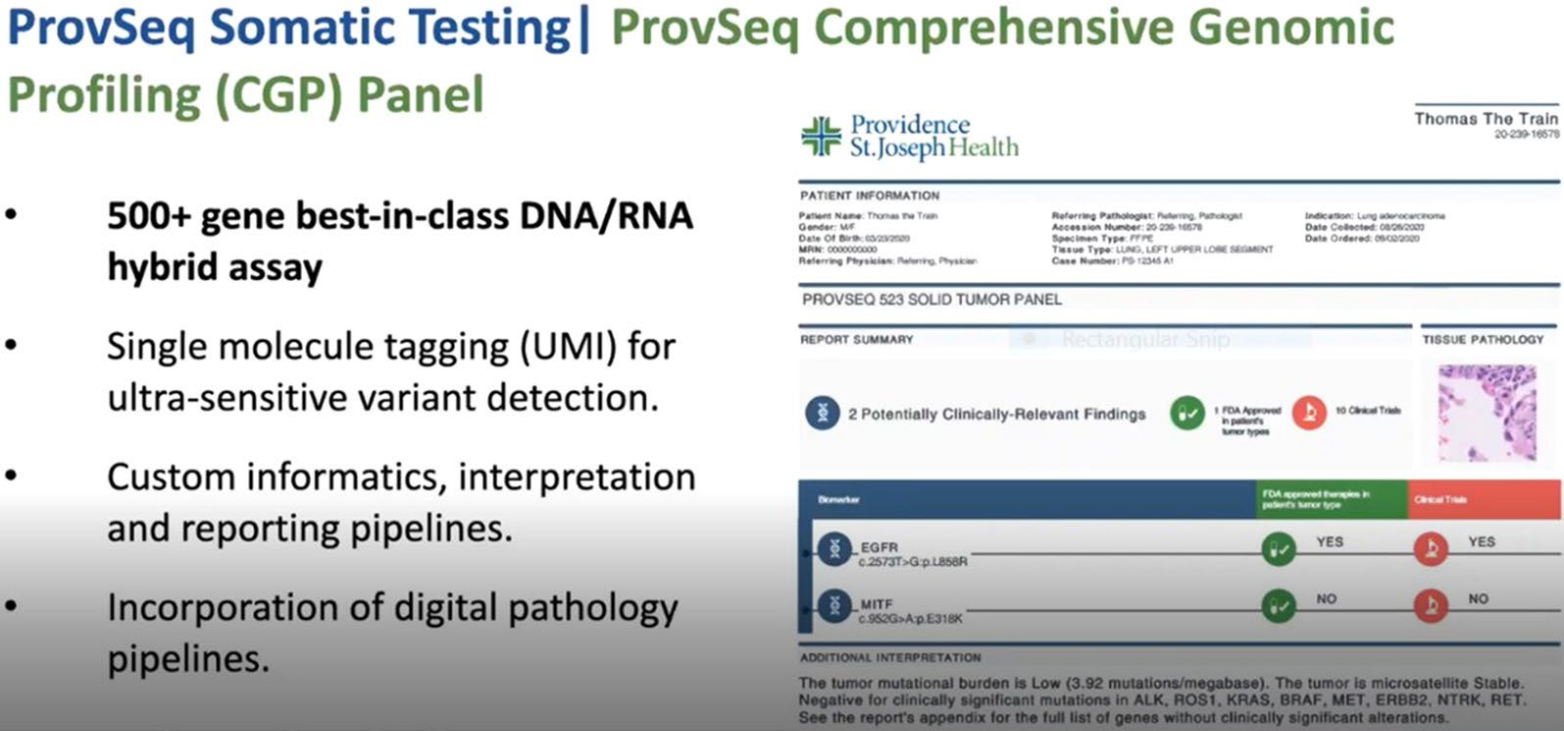
Comprehensive genomic profiling as a standard of care at the Providence Cancer Institute

Despite the significant progress made in integrating this process into clinical routine, translating the gained knowledge into better treatments for patients remains an ongoing challenge. In particular, biomarker based clinical trial matching remains a challenge to scale and timely execute. Attempts to improve in these aspects by developing an ecosystem that empowers the utilization of genomics results are virtual molecular tumor boards, which involves the integration of genomics, imaging, electronic health records and clinical trial matching, and the use of natural language processing and machine learning at scale on electronic medical records to automate the real time identification cohorts of patients eligible for clinical trial participation. Beyond genomics biomarkers, the TME is also a key factor in driving outcomes and therapies, and urgently needs to be integrated into routine pathology workflow, using technologies such as multiplex IHC and IF. In this context, the use of machine learning via deep convolutional neural networks to enable the multiplex IHC/IF-based reverse engineering of hematoxylin and eosin (H&E) images could enable the systematic scaling of the assessment of the tumour microenvironment to large population cohorts and enable to overcome throughput issues existing in current multiplexed staining technological implementations [4, 5] (Fig. 1).

### Leveraging other genomes as potential response biomarkers: the microbiome

Unlike the germline or tumor genome, the gut microbiome is inherently modifiable and is influenced by factors which include diet and medication, as well as anthropometric factors (e.g., body mass index), psychological factors, and geographical location. The gut microbiome has been shown to play a role in the development of mucosal and systemic immunity and there is now considerable evidence that is can be predictive of response to immunotherapy. Several clinical studies have demonstrated a strong association between the gut microbiome and response to immune checkpoint inhibition across different cancer types.

In a study of patients with melanoma undergoing anti-PD-1 therapy, characteristics of the gut microbiome at baseline were associated with response to treatment, with alpha diversity scores significantly higher in responders compared to non-responders [6]. Microbiome compositional features were also associated with response, with a higher relative abundance of bacteria within the *Clostridiales* order (*Ruminococcaceae* family, *Faecalibacterium* genus) in responders and a significantly higher abundance of *Bacteroidales* in non-responders. Bacteria associated with response (in the *Clostridiales* order) were also positively correlated with cytotoxic T cells in the TME of patients with available baseline tumor samples.

However, there is little overlap between response-associated taxa across independent cohorts, with studies in patients with melanoma, renal cell carcinoma, and NSCLC demonstrating differential microbiome signatures in responders and non-responders to immune checkpoint blockade. These differences may be driven in part by technical issues, including processing (i.e., sample collection, preservatives, sample storage, DNA extraction) and the sequencing platform used. This may be overcome by the use of whole genome sequencing and metagenomic methods. However, it may also be that a favorable microbiome is in part dependent on context. Habitual diet is a key factor, with a plant-based diet resulting in a microbiome with different characteristics to that seen with a meat-based diet. The microbiome is a complex ecosystem and the function of the microbiome, which is largely shaped by diet, may be more important than its composition.

Defining a microbiome as favorable or otherwise to stratify and select patients for intervention is challenging. Large-scale cohorts are needed with the selection of appropriate patients and donors critical but complex. Diet should be assessed in all observational microbiome cohorts and both habitual diet and baseline microbiota may influence response to microbiome modulation interventions. The method used to modulate the microbiome and plans for maintenance may result in differences in engraftment or durability of engraftment and ultimately response. Another consideration is that the fecal microbiome may not be the most useful read-out, and changes in the fecal microbiome as a biomarker of response to intervention may be misleading. Integrated analysis of ongoing microbiome modulation trials will be critical to inform ideal donor characteristics for fecal microbiota transplantation studies (host vs complete response donor, microbiome profile, etc.), predictors of effective microbiome modulation/engraftment (e.g., baseline microbiome profile, host characteristics) and microbiome changes that correlate with an immune and disease response.

Emerging data have also demonstrated that there are intratumoral bacteria in some cancers and that these show distinct composition depending on histology [7]. In patients with pancreatic adenocarcinoma (PDAC), long-term survivors had distinct tumor microbiomes, with an intratumoral microbiome signature highly predictive of survival [8]. The tumor microbiome can also be modulated, with fecal microbiota transplantation from PDAC patients affecting tumor growth in a murine model, indicating cross-talk with the gut microbiome.

### CD26: a new biomarker?

CD26 is a surface glycoprotein expressed on various cell types, including immune cells, that has several properties that might affect T cell function, including cleavage of chemokines that regulate migration, T cell co-stimulation via caveolin-1, binding of extracellular matrix proteins, and adenosine conversion. CD26 expression correlates with specific CD4 + T cell subsets, with CD26^high^ CD4 + T cells having distinct antitumor and molecular properties relative to other helper subsets. These cells co-secrete effector cytokines, including interleukin (IL)-17, interferon (IFN)-γ, IL-22, and IL-2, produce cytotoxic molecules, and have enhanced memory (long-term persistence and Bcl2 expression). CD26^high^ T cells also persist and regress tumors to a greater extent than other CD4 + T cells in vivo and represent a distinct CD4 + helper population with potent antitumor properties [9]. Better antitumor responses also correlate with an increased presence of CD26 + T cells in the tumor, suggesting a possible significance of this marker in cancer immunotherapy. It has been postulated that CD26 expression may correlate with productive immune responses after checkpoint blockade.

**Fig. 2:**
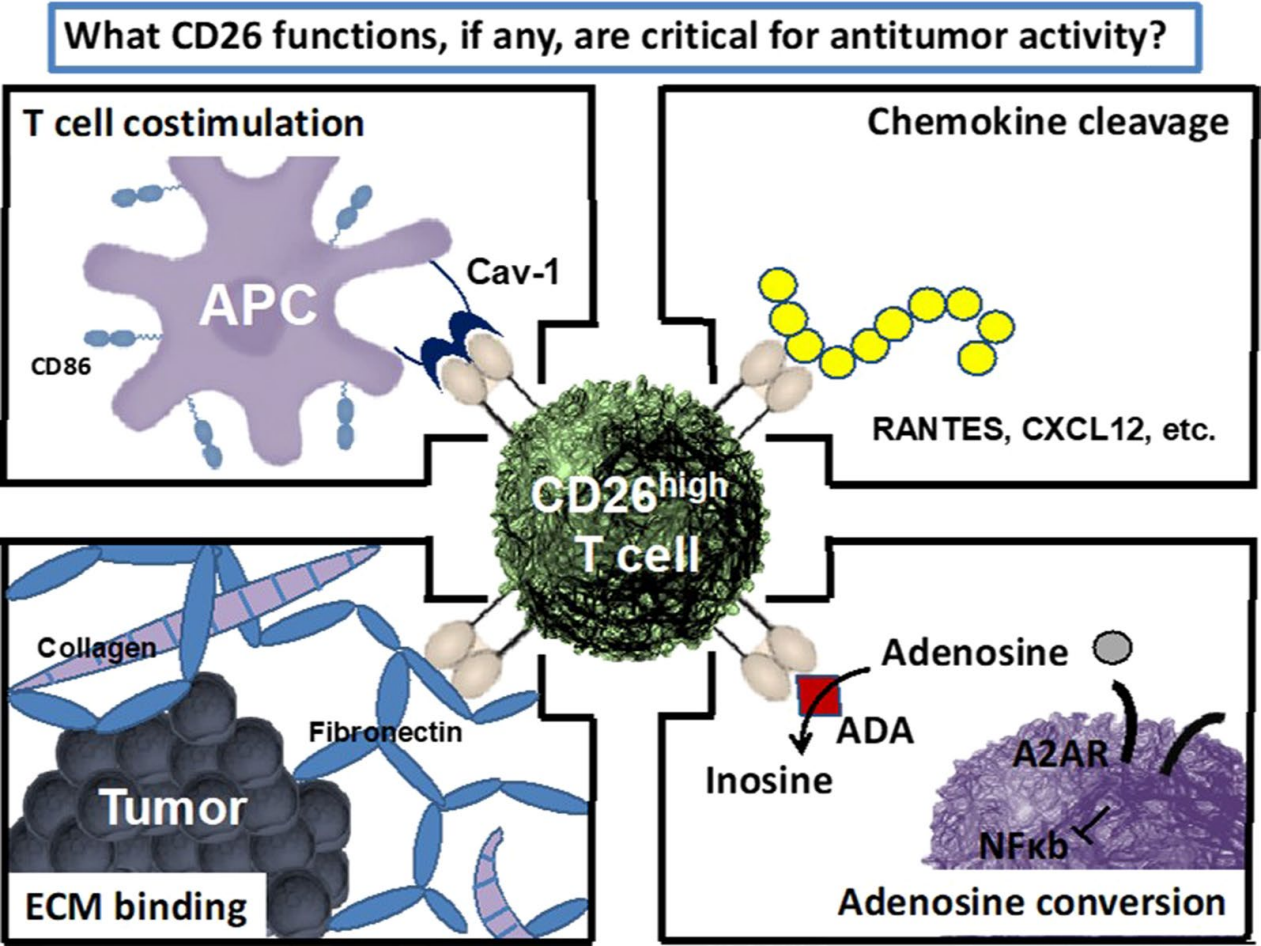
CD26: a new biomarker?

Treatment options for oral cavity squamous cell carcinoma (OCSCC) are limited with 5-year survival of advanced disease of 35–45%. Among patients with platinum-refractory, recurrent squamous-cell carcinoma of the head and neck (HNSCC), treatment with nivolumab resulted in longer overall survival (OS) than treatment with standard, single-agent therapy [10]. Given this, exploring the use of PD-1 therapy in alternate settings for head and neck cancer, such as OCSCC, is warranted and an ongoing phase II trial (NCT03021993) is assessing nivolumab as neoadjuvant therapy for patients with treatment-naïve OCSCC. The first stage of this trial included nine patients with stage II-IVA OSCC and reported a 44% response rate. There was no difference in CD4 and CD8 frequencies between responders and non-responders but a strong trend of more CD26^high^ T cells in responders [11]. Similar trends with CD26^high^ tumor-infiltrating lymphocytes (TILs) have been observed in an ongoing neoadjuvant trial in patients with melanoma. In ten patients with resectable stage IIIB-D melanoma, neoadjuvant nivolumab and pepinemab, a monoclonal antibody to semaphorin 4D that has demonstrated immune cell-dependent, antitumor activity, resulted in a 38% major response rate and increased T cell infiltration [12]. Patients responsive to treatment had more CD26^high^ CD4 + and CD26^high^CD8 + TILs. CD26^high^ CD4 + may have a role as next-generation adoptive cell transfer therapy for non-responders to immune checkpoint blockade (Fig. 2).

### Best of SITC for clinical development and trials

The effects of targeting CD47 and PD-L1 were investigated through syngeneic triple-negative breast cancer (TNBC) murine models and tumor organoid platforms [13]. Targeting CD47 alone or in combination with anti-PD-L1 resulted in decreased tumor burden and increased intratumoral granzyme B secreting CD8 + T cells in a TNBC murine model. Targeting CD47 within organoids increased IFN-γ and granzyme B expression, indicating enhanced CD8 + T cell cytolytic capacity. These data indicate that CD47 targeted monotherapy or combination with anti-PD-L1 may enhance TNBC patient response.

In patients with surgically resectable stage I-IIIA NSCLC, neoadjuvant platinum doublet chemotherapy with nivolumab achieved a more robust tumor and overall pathological downstaging effect and clinically meaningful lower probability of upstaging compared with neoadjuvant nivolumab alone or nivolumab plus ipilimumab [14]. Longer follow-up will be needed to assess whether the downstaging effect results in improved survival. In another trial in the neoadjuvant setting, nivolumab in combination with the virus-like particle-encapsulated toll-like receptor (TLR)-9 agonist CMP-001 had acceptable toxicity and promising efficacy in 30 patients with regionally advanced melanoma [15]. Pathological responses were seen in 70% of patients, with 50% having a complete pathological response (pCR), and was associated with increased CD8 + tumor-infiltrating lymphocytes (TILs) and intratumoral CD303 + plasmacytoid dendritic cells (DCs). In an update to the first clinical trial of an approved oncolytic viral immunotherapy as a neoadjuvant treatment in advanced melanoma, neoadjuvant talimogene laherparepvec (T-VEC) plus surgery resulted in 3-year recurrence-free survival (RFS) of 46.5% vs 31.0% with immediate surgery (hazard ratio [HR] 0.67, P = 0.043) in resectable stage IIIB-IVM1a melanoma [16]. Three-year OS rates were 83.2% for T-VEC plus surgery and 71.6% for surgery alone (HR 0.54, P = 0.061). These data indicate a durable treatment effect of neoadjuvant T-VEC on advanced resectable melanoma.

Eganelisib is a selective PI3K-γ inhibitor that reprograms macrophages and myeloid-derived suppressor cells (MDSCs) from an immunosuppressive to an immune-activating phenotype. In 21 patients with HNSCC treated with eganelisib in combination with nivolumab, overall response rate (ORR) was 10%, the disease control rate (DCR) was 45%, and the clinical benefit rate was 25%; these values were 20%, 30%, and 40%, respectively, in patients that received ≤ 2 lines of prior systemic therapy [17]. The combination of eganelisib and nivolumab also had an acceptable safety profile. AMG 757, a half-life extended bi-specific T-cell engager, binds to DLL3 on tumor cells and CD3 on T cells, resulting in T cell-dependent killing of tumor cells. In an ongoing phase I study in 31 patients with relapsed/refractory small-cell lung cancer (SCLC), AMG 757 had an acceptable safety profile and showed antitumor activity [18]. Overall, 16.1% of patients had grade ≥ 3 treatment-related adverse events. Cytokine release syndrome was the most common adverse event (35.5% of patients), but was mostly grade 1–2, occurred within 24 h of the first or second dose and was reversible. Confirmed partial responses occurred in 16% and stable disease in 26% of all patients. All responders remained on treatment with duration of response (DOR) ranging from 2.0 + to 7.4 + months.

Bemcentinib is a selective AXL kinase inhibitor that has been shown to enhance checkpoint inhibitor efficacy in pre-clinical models. A phase II single-arm study of bemcentinib and pembrolizumab for stage IV NSCLC reported that, among 15 patients who progressed on prior immunotherapy, clinical benefit was shown in 6/7 (86%) AXL-positive patients and none of five AXL-negative patients [19]. Median progression-free survival (PFS) was 4. 7 months in AXL-positive and 1.9 months in AXL-negative patients. The combination was well-tolerated. Transcriptional analysis of pre-treatment biopsies revealed a distinct gene profile correlating with clinical benefit from treatment.

Angiosarcoma is a rare cancer of endothelial cells, a subset of which is characterized by high TMB. In a phase II trial of ipilimumab plus nivolumab which included 16 patients with metastatic or unresectable angiosarcoma, ORR was 25% [20]. Subgroup analysis revealed that 3 of 5 patients with primary cutaneous tumors of the scalp or face had a confirmed objective response. Six-month PFS rate was 38%. The combination of ipilimumab and nivolumab was well tolerated and further investigation in angiosarcoma is warranted.

The benefit of immune checkpoint blockade in patients with leptomeningeal metastases (LMM) is unknown. In a phase II trial in 13 patients with LMM from solid tumors, the majority of which were traditionally responsive to immunotherapy, pembrolizumab resulted in a 38% central nervous system response rate and was well tolerated [21].

## Trends in immunotherapy

### Breast cancer immunotherapy: biomarkers and clinical benefit

TNBC was chosen as the first breast cancer subtype to prioritize for immunotherapy based on a significant unmet clinical need and higher likelihood of tumors being immune-activated. In the IMpassion130 trial, 902 patients with untreated metastatic TNBC were randomized to atezolizumab plus *nab*-paclitaxel or placebo plus nab-paclitaxel until disease progression or unacceptable toxicity. In the final overall survival analysis, there was no significant improvement in OS with atezolizumab vs placebo in the intent-to-treat population (median OS of 21 vs 18.7 months; HR: 0.87 [0.75, 1.02]; P = 0.077) [22]. In patients with PD-L1 immune cell-positive tumours (≥ 1% PD-L1 expression), median OS was 25.4 months with atezolizumab vs 17.9 months with placebo; the HR was 0.67 but statistical significance was not formally tested as per the prespecified testing hierarchy. Three-year OS rates were 36% vs 22%, respectively. The combination of atezolizumab plus *nab*-paclitaxel remained safe and tolerable with no new safety signals, and these results support a positive benefit-risk profile in patients with PD-L1 immune cell-positive TNBC.

In a second trial, IMpassion 131,651 patients with metastatic or unresectable locally advanced TNBC were randomized to first-line atezolizumab plus paclitaxel or placebo with paclitaxel. In contrast to the OS benefit shown for atezolizumab plus *nab*-paclitaxel in the IMpassion130 trial, PFS was not significantly improved by atezolizumab plus paclitaxel vs paclitaxel with placebo in either the PD-L1-positive (6.0 vs 5.7 months; HR = 0.82; P = 0.20) or the intent-to-treat population (5.7 vs 5.6 months; HR = 0.86; significance not formally tested due to the hierarchical statistical analysis plan) [23]. OS was also not improved with atezolizumab, either in the PD-L1–positive (HR = 1.12) or the intent-to-treat patient populatiosn (HR = 1.11). Potential reasons for the difference in results between IMpassion 130 and IMpassion 131 require further exploration.

In an exploratory biomarker analysis of IMpassion 130, PFS and OS were evaluated based on PD-L1 expression on immune cells and tumor cells, intratumoral CD8, stromal TILs, and BRCA1/2 mutations [24]. The majority of patients with PD-L1 expression in tumor cells were included within the PD-L1 immune cell-positive population. Consistent clinical benefit of atezolizumab plus *nab*-paclitaxel was seen for PD-L1 immune cell-positive patients using different cut-off values, provided those cells occupied at least 1% or more of the tumor area (Table 1). Intratumoral CD8 and stromal TIL positivity were associated with PD-L1 immune cell-positive status and only predicted a benefit with treatment in patients who were also PD-L1 immune cell-positive. PD-L1 immune cell-positive patients benefited from treatment regardless of BRCA1/2 mutation status.

**Table 1:** Exploratory biomarker analysis of IMpassion 130

Biomarker	Number of subjects	HR PFS A + nP vs P + nP	mPFS	HR OS A + nP vs P + nP	mOS
PD-L1 immune cell expression ≥ 1% and < 5% (low)	243/902(26.9%)	0.61 (95% CI 0.46–0.80) p < 0.005	7.4 mo	0.68 (95% CI 0.48–0.94) p = 0.02	22.6 mo
PD-L1 immune cell expression ≥ 5% (high)	125/902 (13.9%)	0.71 (95% CI 0.48–1.05) p = 0.09	9.3 mo	0.76 (95% CI 0.46–1.26) p = 0.29	28.9 mo

A total of 902 patients were enrolled and randomized equally to receive either A + nP or P + nP. 40.8% of patient enrolled in the trial were PD-L1 immune cell-positive, defined as PD-L1-positive immune cells occupying at least 1% of the tumor area as determined by the Ventana SP142 assay. Data from Emens JNCI 2021

*HR* hazard ratio, *A* atezolizumab, *nP* nab-paclitaxel, *P* placebo, *PFS* progression-free survival, *mPFS* median progression-free survival, *OS* overall survival, *mOS* median overall survival, *PD-L1* programmed death ligand-1, *CI* confidence interval, *mo* months

Pembrolizumab has also been assessed in combination with chemotherapy in the KEYNOTE 355 trial, in which 847 patients with metastatic TNBC were randomized to pembrolizumab plus chemotherapy (*nab*-paclitaxel, paclitaxel, or gemcitabine plus carboplatin) or placebo plus chemotherapy [25]. The addition of pembrolizumab was associated with a significant improvement in PFS in patients with PD-L1 expression (combined positive score ≥ 10), suggesting a clinically meaningful role for pembrolizumab in combination with chemotherapy as first-line treatment.

### Immunotherapy for human papillomavirus-related head and neck cancer

In patients with HNSCC, high density of tumor-infiltrating CD8 T cells in the TME is associated with better survival. Human papillomavirus (HPV)-positive HNSCC is more likely to exhibit an immunologically active TME with more PD-1-positive CD8 T cells than HPV-negative disease, highlighting the potential for improved activity in this population. Acute and longer-term toxicity is increasing in a younger group of HPV-positive HNSCC patients, which warrants re-evaluation of the conventional chemoradiation-based therapeutic approach in this cohort.

In the CheckMate 141 trial, patients with platinum-refractory, recurrent HNSCC treated with nivolumab had improved OS compared to patients treated with standard, single-agent therapy of methotrexate, docetaxel, or cetuximab [10]. Long-term OS was similar in patients with and without PD-L1 expression, although responses occurred earlier in PD-L1-positive patients [26]. OS was also similar irrespective of HPV status, with an approximately 40% reduction in risk of death in HPV-positive and HPV-negative patients. However, responses were more frequent and again occurred earlier in HPV-positive patients.

In a neoadjuvant setting, treatment with two doses of nivolumab resulted in tumor reduction in HPV-positive and HPV-negative patients in the CheckMate 358 trial. HPV-positive patients had better RFS than HPV-negative patients, with 2-year survival of over 90%, which is typical for this cohort. Pre-operative immunotherapy could potentially reduce the extent of surgery while post-operative adjuvant use could improve RFS and replace chemotherapy.

Assessment of transcriptional profiles of single cells from peripheral and intratumoral immune populations from patients with HNSCC showed that TILs from HPV-positive tumors had distinct features and unique receptor-ligand interactions, especially in T follicular helper cells and germinal center B cells [27]. In addition, a higher elastic cancer-associated fibroblast score was significantly associated with worse OS in HPV-positive patients and this is an area of further research. These emerging unique features of HPV-positive HNSCC may help explain better prognosis but are not yet predictive for immunotherapy.

In the placebo-controlled JAVELIN Head and Neck 100 trial, 697 patients with histologically confirmed, previously untreated HNSCC of the oropharynx, hypopharynx, larynx, or oral cavity were randomized to concurrent chemoradiation with or without avelumab. In an interim analysis, PFS and OS were both in favor of the chemoradiation alone group, with the addition of avelumab providing no benefit regardless of HPV status [28]. In an ongoing trial, concurrent chemoradiation and pembrolizumab is being compared with sequential chemoradiation followed by pembrolizumab. In another trial, nivolumab is being combined with de-escalated radiation in patients with intermediate risk p16 + oropharyngeal cancer.

### Targeting the cGAS-STING pathway within tumor-associated macrophages to enhance immune responsiveness in prostate cancer

Multiple mechanisms of intrinsic resistance to immunotherapy in prostate cancer include paucity of immune cell infiltrate, which is primarily composed of myeloid immunosuppressive cells, a highly immunosuppressive cytokine milieu, low TMB, downregulation of major histocompatibility complex (MHC) class I, and compensatory feedback immune checkpoint expression in response to checkpoint inhibitor treatment.

In recent years, the DNA sensing cGAS/STING pathway has emerged as a therapeutic strategy to treat cancer. Furthermore, reprogramming of myeloid suppressive cells, such as macrophages and myeloid-derived suppressor cells, have been investigated in preclinical murine prostate cancer models. Given the multiple mechanisms of resistance to immunotherapy in prostate cancer, rational immuno-oncology combination strategies that activate both innate and adaptive immunity in prostate cancer, with a focus of on activation of c-GAS/STING signaling within the tumor microenvironment, carries the potential to enhance therapeutic efficacy.

### Adaptive metabolic rewiring of the tumor microenvironment impedes efficacy of IDO blockade in ovarian cancer

Indoleamine 2,3-dioxygenase (IDO) is a potent mechanism of immune tolerance through its involvement in tryptophan catabolism, which leads to T-cell anergy and apoptosis and has an important role in suppressing antitumor immune responses in cancer. IDO results in local depletion of tryptophan, and accumulation of tryptophan catabolites, including kynurenine and its derivatives, depending on the presence of downstream enzymes in the kynurenine pathway. IDO functional activity has been associated with worse outcomes in patients with ovarian cancer, suggesting reduced IDO enzyme activity might be associated with clinical benefit. In a murine model, tumor-derived IDO was associated with poor OS and a reduction in intratumoral CD8 + T cells. These data suggest that IDO inhibition may potentially synergize with PD-1 inhibition. The first-in-human study with the IDO1 inhibitor epacadostat did not result in objective responses in patients with advanced solid tumours; however, when combined with nivolumab or pembrolizumab, approximately 60–65% of patients had an objective response. In a subsequent phase III trial, no significant survival advantage was observed with the addition of epacadostat to pembrolizumab in patients with advanced melanoma [29]. One potential reason for this lack of efficacy may be adaptive metabolic rewiring.

To assess whether IDO1 inhibition might decrease immune suppression and increase CD8 + TILs, a pilot study in which patients with epithelial ovarian, fallopian tube or primary peritoneal carcinoma received neoadjuvant epacadostat was conducted. Treatment with epacadostat resulted in a decrease in kynurenine and the kynurenine:tryptophan ratio in both plasma and the TME, indicating treatment was effective in reducing tryptophan catabolism. Metabolic adaptation beyond blockade of kynurenine was observed, with increases in metabolites in several pathways, including nicotinamide, serotonin, purine, and others. This indicates a metabolic switch in the TME with the emergence of alternative pathways for tryptophan catabolism following IDO blockade. Epacadostat also drove changes in the transcriptional signature indicating enrichment of tryptophan catabolism alternative pathways.

Together, alteration of the kynurenine:tryptophan ratio in the TME by epacadostat induces a unique gene and metabolic signature, that may explain the lack of additional benefit of IDO inhibition with immune checkpoint blockade in phase III trials. These data provide a rationale to explore concomitant inhibition of local suppressive metabolites and IDO1, in order to overcome the detrimental metabolic switch in the TME.

### Immunotherapy in GI Cancer

Colorectal cancer is a highly heterogenous disease and an active immune response is limited to subgroups of patients. Currently, the only effective immunotherapies are obtained in molecularly selected MSI-high (MSI-H) or mismatch repair-deficient (dMMR) tumours. The question is whether it is possible to activate immune competence in microsatellite-stable (MSS) tumours.

Clinical studies have shown that mismatch-repair deficiency predicted clinical benefit of immune checkpoint blockade with pembrolizumab in treatment-refractory patients with colorectal cancer and across several other tumor types [30, 31]. First-line treatment with nivolumab in combination with low-dose ipilimumab also resulted in durable responses and disease control in patients with MSI-H/dMMR metastatic colorectal cancer in the phase II CheckMate 142 trial [32]. In an updated analysis of this study with a median follow-up of 13.8 months, the combination resulted in an ORR of 64%, a complete response rate of 9%, and a DCR of 84%, indicating significant antitumor activity [33]. Treatment- was well tolerated with treatment-related grade 3–4 toxicities reported in 20% of patients and only two patients (4%) discontinuing therapy because of a treatment-related adverse event. Pembrolizumab also showed improved efficacy as first-line treatment vs chemotherapy in a phase III trial of 307 patients with metastatic MSI-H/dMMR colorectal cancer [34]. Patients receiving pembrolizumab had a median PFS of 16.5 months vs 8.2 months with chemotherapy (HR = 0.60; p = 0.0002). Pembrolizumab was also associated with fewer grade ≥ 3 treatment-related adverse events, which occurred in 22% of patients compared to 66% of the chemotherapy group.

In the phase III IMblaze 370 trial, 363 patients with unresectable locally advanced or metastatic colorectal cancer and disease progression or intolerance to at least two previous systemic chemotherapy regimens were treated with atezolizumab plus cobimetinib, atezolizumab monotherapy or the multi-kinase inhibitor regorafenib [35]. Patients with MSI-H tumors were limited to approximately 5% of the cohort. The trial failed to reach its primary endpoint of improved OS with atezolizumab plus cobimetinib or atezolizumab vs regorafenib (median OS of 8.8 months with atezolizumab plus cobimetinib, 7.1 months with atezolizumab, and 8.5 months with regorafenib). Lack of clinical activity may be due to the immune-excluded phenotype of metastatic colorectal cancer, and simultaneous PD-1 blockade and mitogen-activated protein kinase (MAPK)-mediated immune suppression may not be sufficient to generate antitumour immune responses in immune-excluded tumours. Efficacy in MSI-high disease could not be estimated due to low patient numbers.

Another trial has assessed a rechallenge strategy with avelumab plus the epidermal growth factor receptor inhibitor cetuximab. Given that cetuximab enhances antibody-dependent cellular cytotoxicity (ADCC) and promotes expression of MHC class II molecules on DCs, combining with avelumab may be a relevant rechallenge strategy in RAS wild-type metastatic colorectal cancer. Preliminary analysis of the single-arm phase II CAVE mCRC study suggested avelumab plus cetuximab as a rechallenge strategy is effective and well tolerated in patients with chemorefractory RAS/BRAF wild-type metastatic colorectal cancer [36].

### Merkel cell carcinoma

Merkel cell carcinoma (MCC) is a rare aggressive skin cancer linked to ultraviolet light exposure and the Merkel-cell polyomavirus (MCPyV) that is associated with poor survival. Although chemosensitive, responses are rarely durable.

In a preliminary phase II trial, first-line therapy with pembrolizumab in 26 patients with advanced MCC resulted in an ORR of 56% [37]. This study was subsequently expanded to 50 patients; ORR was also 56% and median DOR was not reached after a median follow-up of 15 months [38]. Two-year PFS and OS rates were 48.3% and 68.7%, respectively, and OS was favorable compared with historical chemotherapy controls. Neither PFS nor OS correlated with MCPyV status. Durable responses were also observed with nivolumab in a phase II trial of 25 treatment-naïve or treatment-experienced patients [39].

The anti-PD-L1 avelumab was associated with durable responses and was well tolerated in the phase II JAVELIN Merkel 200 trial of patients with chemotherapy-refractory, advanced MCC [40]. After a median follow-up of 40.8 months, ORR was 33.0 and median DOR was 40.5 months [41]. Median OS was 12.6 months and the 42-month OS rate was 31%. There was a trend towards higher ORR in patients with a higher TMB and, among high TMB patients, the highest response rates were in patients who were PD-L1-positive or MCPyV-negative. High MHC class I expression was also associated with trends for improved ORR and OS. Avelumab also resulted in good response rates when used as first-line therapy in patients with metastatic MCC [42]. Data from these trials suggest PD-1/PD-L1 inhibition may represent a new standard of care in advanced MCC. The avelumab expanded access program for patients with metastatic MCC demonstrated efficacy and safety in a real-world setting, with an ORR of 47% in 240 evaluable patients and no new safety signals [43].

Trials in the neoadjuvant and adjuvant setting in patients with MCC are ongoing. In the first neoadjuvant trial of checkpoint inhibitors in MCC, nivolumab administered approximately 4 weeks before surgery was generally well tolerated and induced pCRs and radiographic tumor regressions in 17 of 36 treated patients [44]. Responses were observed regardless of tumor MCPyV, PD-L1, or TMB status.

Another possible development is combination therapy. Durable remission after rechallenge with ipilimumab and nivolumab has been reported in metastatic MCC refractory to avelumab [45, 46]. The class I histone deacetylase (HDAC) inhibitor domatinostat exerts direct antitumoral effects and restores human leukocyte antigen (HLA) class I surface expression on MCC cells [47], which may increase reverse resistance to immunotherapy. The MERKLIN2 trial of domatinostat in combination with avelumab in patients with advanced MCC who have progressed on anti-PD-(L)1 is currently recruiting. Finally, the combination of avelumab with low to moderate-dose chemotherapy and an immune enhancer such as IL-15 superagonist N-803 resulted in a complete response in an MCC patient in whom avelumab monotherapy was ineffective [48].

## Drivers in immune responses

### Mechanisms of immunotherapy response and resistance

The efficacy of immunomodulatory agents depends on the presence of a baseline adaptive immune response and pre-existing immunity being utilized via inhibition of checkpoint receptors on T cells. Tumors may be categorized as hot, altered (immune-excluded or immuno-suppressed) or cold, based on their immune contexture [49]. Hot immune tumours have high Immunoscore, checkpoint activation or otherwise impaired T cell functions. Altered-immunosuppressed immune tumours have an intermediate Immunoscore, and the presence of soluble inhibitory mediators, immune suppressive cells (MDSCs and regulatory T cells), and T cell checkpoints. Altered-excluded immune tumours have no T cell infiltration inside the tumour bed, intermediate Immunoscore, activation of oncogenic pathways, epigenetic regulation and reprogramming of the TME, aberrant tumour vasculature and/or stroma, and hypoxia. Cold immune tumours have low Immunoscore and failed T cell priming [50].

A key question is whether adoptive cellular therapy can overcome failed spontaneous T cell priming and convert cold into hot tumours [51, 52]. The ZUMA-1 trial demonstrated a high rate of durable response and a manageable safety profile with axicabtagene ciloleucel (axi-cel), an anti-CD19 chimeric antigen receptor (CAR) T-cell therapy, in patients with refractory large B-cell lymphoma [53]. Univariate and multivariate analyses indicated that rapid CAR T-cell expansion commensurate with pretreatment tumor burden (influenced by product T-cell fitness), the number of CD8 and CCR7 + CD45RA + T cells infused, and host systemic inflammation, were the most significant determining factors for durable response [54]. Tumor immune microenvironment-mediated suppression may also have been an important factor in determining response and pre-existing T cell-involved features of the TME (high Immunoscore, High Immunosign) may be associated with a response to CAR-T cell therapy. In analysis of TME factors, CAR T cell therapy responders had elevated pretreatment TME Immunosign21 scores compared to non-responders [55]. Higher pretreatment Immunoscore and pretreatment intra-tumor densities of CD3 + and CD8 + T cells were all positively associated with a complete response [56]. TME gene expression analysis suggested a pan-inflammatory profile, including myeloid- and DC-related gene expression, in patients who achieved a complete response with higher Immunosign^®^ 21 and Immunoscore. These data indicate that a stronger immune contexture predicts an increased likelihood of response, supporting the idea that CAR-T cell therapy alone may not be sufficient to treat patients with cold tumours, other than in a subset of patients with low disease burden. These findings support the need for the development of anti-CD19 CAR T cell treatment optimizations designed to overcome an immune-detrimental TME.

### Breast cancer, radiation and immunotherapy

Radiation results in immunogenic cell death and facilitates tumor neoantigen presentation and cross-priming of tumor-specific T cells, turning the irradiated tumor into an in situ vaccine. However, established tumors have in place multiple immune escape mechanisms that generally offset the capacity of radiotherapy alone to result in a systemic response of metastatic disease sites (abscopal effect). Moreover, radiation also elicits immune suppressive signals, like activation of transforming growth factor (TGF) β. Various strategies have been used to try and shift the balance from immunosuppressive to pro-immunogenic signals of radiation, either by offsetting negative effects (e.g., by blockade of cytotoxic T-lymphocyte-associated antigen [CTLA]-4, transforming growth factor [TGF]-β, CD73, PDL-1, or VISTA) or be enhancing positive effects (e.g., by using TLR agonists, DC growth factors, IFN inducers, IL-15, etc.).

An alternative approach is to attempt to optimize the immunogenicity of radiation when combined with chemotherapy or endocrine therapy. There is data to suggest that the aromatase inhibitor letrozole may have an indirect antitumor mechanism of action through reducing regulatory T lymphocytes (Tregs) in breast tumors [57] and that cyclin-dependent kinase 4/6 (CDK4/6) inhibitors have multiple immunological effects in estrogen receptor (ER) + breast cancer [58]. Thus, the beneficial effects on survival in breast cancer reported with aromatase inhibitors and the CDK4/6 inhibitors palbociclib and ribociclib is likely to also be mediated by their respective immune effects. We hypothesized that these immune-modulating effects could be enhanced by radiation and investigated preclinically whether survival could be improved by combining radiation therapy with aromatase inhibitors and CDK4/6 inhibitors.

In a novel syngeneic preclinical model of ER + mammary carcinoma [59] efficacy of radiotherapy combined with CDK4/6 inhibitor palbociclib and tamoxifen were investigated in various doses and therapeutic schedules [60]. In vitro, radiotherapy and palbociclib administered as standalone agents had partial cytostatic effects, correlating with suboptimal tumor control in vivo. However, while palbociclib + tamoxifen delivered before focal radiation provided minimal benefit compared with each treatment alone, delivering palbociclib + tamoxifen after focal radiotherapy mediated superior therapeutic effects, with mice receiving radiotherapy followed by palbociclib + tamoxifen having the best survival outcomes. Preliminary single cell analysis experiments demonstrate different immunological profiles in the different sequencing groups and suggest that radiation followed by palbociclib + tamoxifen reduces immunosuppressive barriers. This data was translated to a prospective randomized trial in metastatic ER + breast cancer patients, comparing etrozole + palbociclib to the same regimen preceded by stereotactic body radiation therapy to up to five metastases (NCT04563507).

### Immunotherapy of lymphomas

Antibodies targeting CD20 have likely reached a plateau in efficacy, with newer generation anti-CD20 antibodies having generally similar efficacy to rituximab. In patients with relapsed follicular non-Hodgkin lymphoma (NHL), obinutuzumab, which is engineered for enhanced ADCC and pro-apoptotic activity, did not improve PFS vs rituximab [61]. CD19 antigen represents another target and tafasitamab, a humanized anti-CD19, demonstrated clinical activity as a monotherapy in relapsed or refractory B cell NHL and in combination with lenalidomide in patients with relapsed or refractory diffuse large B cell lymphoma (DLBCL) [62, 63].

Checkpoint inhibitors in NHL are typically associated with low response rates, with PD-1 blockade only appearing to be useful in rare NHL subtypes, including primary mediastinal B cell lymphoma, NK/T cell lymphoma, or Richter transformation of chronic lymphocytic leukemia. The highest response rates to PD-1 blockade are in refractory Hodgkin lymphoma. Among patients with recurrent classical Hodgkin lymphoma who failed to respond to autologous stem-cell transplantation and had either relapsed after or failed to respond to brentuximab vedotin, nivolumab resulted in a 66% ORR [64]. Similarly, pembrolizumab resulted in a high response rate in patients with relapsed or refractory classical Hodgkin lymphoma [65]. However, most lymphoma patients ultimately have disease progression after PD-1 blockade. The combination of dual PD-1 and CTLA-4 blockade (nivolumab plus ipilimumab) in patients with relapsed or refractory lymphoid malignancies had no meaningful improvement in efficacy vs single-agent nivolumab [66].

Three anti-CD19 CAR T cell therapies are now approved for use in NHL, the 41BB-containing tisagenlecleucel and the CD-28 based axicabtagene ciloleucel and lisocabtagene ciloleucel. These therapies have achieved high response rates in DLBCL, ranging from 52% with tisagenlecleucel to 82% with axicabtagene ciloleucel, and these responses can be durable [53, 67, 68]. However, CD28 CAR T cells appear more toxic than 41BB CAR T cells, but both carry risk of neurotoxicity and more studies are needed to address this question. Side effects of CAR T cell therapy can be severe, life-threatening, and limits who is eligible to receive this therapy, with up to one-third of patients requiring intensive care unit admission. Mechanisms of anti-CD19 CAR T resistance include loss of CD19 and antigen escape (CD19 alternative splicing, CD19 mutation) and impaired T-cell fitness (e.g., due to the apheresis and/or CAR T product, host environment or TME). Various strategies to improve the efficacy of CAR T therapy in B-cell malignancies through targeting multiple antigens (e.g., CD19-CD22 or CD19-CD20 CAR T) and improving T-cell fitness are under investigation. In a recent trial, CD19 CAR T therapy was also shown to be active in mantle-cell lymphoma, with treatment resulting in durable remissions in a majority of patients with relapsed or refractory disease [69].

Another approach involves bispecific antibodies targeting CD20.These are antibody-based molecules engineered to bind two different epitopes, one targeting tumor cells and the other one effector cells, usually T-lymphocytes. Examples of these include blinatumomab, mosunetuzemab, and REGN1979. These off-the-shelf agents may be useful for patients who are unable to tolerate, wait for, or afford CAR T cell therapy.

### Revolt of the T cell system against anti-PD-1 immunotherapy

Patients who respond to PD-1 checkpoint blockade are generally those with either high immune T cell infiltration or those with high TMB, both of whom have primed or activated CD8 T cells. This raises the question of whether resistance can be reversed with proper priming of T cells. In anti-PD-1-resistant models, simultaneous anti-PD-1 and cancer vaccine therapy reversed resistance with reduced tumor volume and improved survival. However, PD-1 blockade prior to antigen priming with cancer vaccine results in impaired antigen-specific CD8 + T cells tumor-infiltration and abrogates the antitumor immune effect [70]. PD-1 blockade prior to antigen priming results in apoptosis of CD8 + T-cells and prevents CD8 + T-cell activation.

Blockade of PD-1 leads to a significant decrease in phosphorylation of SHP2 and release of downstream signaling. Blockade of PD-1 before peptide stimulation led to a significant decrease in phosphorylation of SHP2 while enhancing phosphorylation of Lck and tyrosine-protein kinase ZAP-70 (Zap70). Despite further decreases in phophosphorylated-SHP2, Lck and Zap70 phosphorylation was significantly reduced with the subsequent addition of anti-PD-1. Moreover, the kinase activity of Zap70 was significantly reduced when cells were treated with anti-PD-1 before peptide stimulation. Thus, simultaneous treatment with anti-PD-1 and antigen priming induces T cells that maintain their functional status. However, PD-1 blockade before priming drives T cells into a non-responsive state. LAT and Akt do not get phosphorylated, leading to dysfunctional CD8 T cell production.

These dysfunctional CD8 T cells express both PD-1 and CD38. PD-1 + CD38^hi^ CD8 + T cells fail to respond to antigenic stimulation and do not elicit effector functions. PD-1 blockade before antigenic priming led to a significant increase in the number of PD-1 + CD38^hi^ total and antigen-specific CD8 + T cells. However, simultaneous PD-1 blockade and cancer vaccine resulted in a significant decrease in the number of PD-1 + CD38^hi^ CD8 + T cells. PD-1 + CD38^hi^ cells induced as a result of PD-1 blockade pretreatment were dysfunctional since they failed to upregulate CD40L and did not produce IFN-γ after antigenic restimulation. PD-1 blockade on sub-optimally primed CD8 + T cells induced dysfunctional PD-1 + CD38 CD8 + T cells both in vivo and in vitro. Opposing cytokines were upregulated in PD-1 + CD38^hi^ CD8 cells, with both inflammatory and inhibitory cytokines being released. Consensus hierarchical analysis also showed a distinct clustering of genes related to both cell exhaustion and effector functions.

In baseline or post-treatment tumor biopsies and peripheral blood mononuclear cells (PBMCs) from metastatic melanoma patients, numbers of PD-1 + CD38^hi^ CD8 + T cells correlated with the anti-PD-1 therapeutic response, High numbers of dysfunctional CD8 + T-cells in the tumors and PBMCs served as a predictor of failure of anti-PD-1 therapy. Anti-CD38 antibody treatment may prevent induction of dysfunctional PD1 + CD38^hi^ CD8 + T-cells in the TME and may reverse anti-PD-1 resistance.

### Transcriptional imprints of inherited T-cell regulome modulating immunotherapy outcomes

Several biomarkers have been suggested for predicting response to immune checkpoint inhibitor therapy. However, the predictive capacity of current biomarkers is limited, with significant heterogeneity of outcomes at the level of the individual patient, and there is a need for more personalized biomarkers.

Germline genetics effects host immunity, with genetic factors explaining the large variance in the abundance and activation state of multiple immune cell types, including CD4 + and CD8 + T cells, immunomodulatory molecules, and immune-related genes. In fact, > 70% of T-cell specific variation may be explained by cis-acting inherited genetic variation [71]. As such, germline genetic factors impact on the efficacy and toxicity of checkpoint inhibitor therapy and offer potential as personalized biomarkers. Genetic risk loci for auto-immune and inflammatory diseases have been identified in genome-wide association studies. These have also revealed that specific immune-cell phenotypes, such as T helper cells, CD4 + and CD8 + T cells, have a consistent enrichment for autoimmunity risk variants [72].

Despite considerable effort, evidence supporting the biological relevance of associated germline variants remains elusive, as they map almost exclusively in non-coding regions. Findings from genome-wide association studies estimate that 88% of disease/trait-associated germline variants are non-coding. Genetic variants identified to date for associations with melanoma risk or prognosis almost entirely map to non-coding regions with unknown biological impact. As such, the non-coding regulome may be important in cytotoxic CD4 and CD8 T cell status and immune checkpoint inhibitor response.

Assessment of immunoregulatory pathways using genome-wide maps of expression quantitative trait loci (eQTL) revealed lymphocyte-specific eQTLs that were associated with better OS in patients with cutaneous melanoma [73].

A non-coding genetic variation that is associated with melanoma survival is enriched in open chromatin and transcriptome in CD8 + T cells in patients treated with anti-PD-1 antibodies. Our data indicate the CD8 + specific signatures of 36 genes controlled by seven transcription factors, significantly enriched by autoimmune genetic susceptibility. By developing a novel platform that integrates transcriptomics, open chromatin assessment and whole genome sequencing data, the genetic underpinning of transcriptional regulatory networks of CD8 + T cells associated with immune checkpoint inhibitor response, efficacy and toxicity was identified. Extensive analysis based on well-curated specimens and clinical trial data is ongoing.

### T cell differentiation states in the irradiated tumor microenvironment that drive responses to CTLA-4 blockade

Pre-clinical and clinical evidence supports the ability of focal tumor radiotherapy to enhance responses to immunotherapy. In murine tumor models, a key mechansism underlying the ability of radiation to induce local and systemic responses to anti-CTLA-4 in tumors resistant to anti-CTLA4 alone is the induction of IFN type I [74]. This effect is achieved through accumulation of cytosolic DNA that activates the cGAS/STING pathway and leads to the intratumoral accumulation of Batf3-dependent DCs and the priming of CD8 + T cells [75]. In patients with chemo-refractory metastatic NSCLC, the combination of radiation therapy and CTLA-4 blockade induced systemic antitumor T cells when anti-CTLA-4 antibodies had failed to show efficacy alone or in combination with chemotherapy [76]. Increased serum IFN-β after radiation and early dynamic changes of blood T cell clones were the strongest predictors of response. Functional analysis in one patient with a complete response showed the rapid in vivo expansion of CD8 + T cells that recognized a neoantigen upregulated by radiation.

We are currently investigating the characteristics of an effective anti-tumor T cell response generated by radiation and anti-CTLA4 in mice. We have previously shown that treatment with radiotherapy and anti-CTLA-4 in combination increased TIL density and CD8/CD4 ratio [77]. Radiation increased the clonality and divergence of T cell receptor (TCR) repertoire when used in combination with anti-CTLA-4, suggesting a diverse TCR repertoire is required to achieve tumor rejection and may underlie the synergy between radiotherapy and CTLA-4 blockade.

Our recent data from single cell analysis indicate that together, radiotherapy and CTLA-4 blockade cause a shift in the functional state of tumor-specific CD8 + T cells from cytotoxic to cytokine producers (Rudqvist et al., submitted), and suggest that the combined activity of a range of differentiation states within the T cell compartment is required for tumor control.

## Conclusions

Immunotherapy is now a critical element in the treatment of an increasing number of tumor types, and in many situations has become a new standard of care. Many patients who previously had limited treatment options are now benefiting from advances in our understanding of the TME and immune response with immunotherapies that offer durable responses and improved survival.

However, immunotherapy remains ineffective or suboptimal in many cases and there is a need to further expand the range of patients who achieve a durable benefit. Various strategies to achieve this goal are being explored, including the development of new treatments and the combination of these and existing treatments in novel combination approaches. The efficacy of immunotherapy is largely dependent on the existence of a baseline adaptive immune response and efforts are focused on shifting the balance from an immunosuppressive TME to an immuno-activated contexture. The development of effective biomarkers to guide immunotherapy and better integration of the identification of these into current work processes is another focus of research and should help ensure that patients are treated with the most appropriate option.

Immunotherapy has revolutionized the treatment of many cancers and provided a long-term survival benefit for many patients. Insights from ongoing research and further collaborative efforts, such as those summarized at this Immunotherapy Bridge, should help to continue this progress.

## Data Availability

Not applicable.

## Abbreviations

ADCC: Antibody-dependent cellular cytotoxicity
CAR: Chimeric antigen receptor
CDK: Cyclin-dependent kinase
CTLA-4: Cytotoxic T-lymphocyte-associated antigen-4
DC: Dendritic cell
DCR: Disease control rate
DLBCL: Diffuse large B cell lymphoma
dMMR: Mismatch repair-deficient
DOR: Duration of response
eQTL: Expression quantitative trait loci
ER: Estrogen receptor
FDA: Food and Drug Administration
HDAC: Histone deacetylase
H&E: Hematoxylin and eosin
HLA: Human leukocyte antigen
HNSCC: Head and neck squamous cell carcinoma
HPV: Human papillomavirus
HR: Hazard ratio
HRP: Horseradish peroxidase
IDO: Indoleamine 2,3-dioxygenase
IF: Immunofluorescence
IFN: Interferon
IHC: Immunohistochemistry
IL: Interleukin
LMM: Leptomeningeal metastases
MAPK: Mitogen-activated protein kinase
MCC: Merkel cell carcinoma
MCPyV: Merkel-cell polyomavirus
MDSC: Myeloid-derived suppressor cells
MHC: Major histocompatibility complex
MSI: Microsatellite instability
NHL: Non-Hodgkin lymphoma
NSCLC: Non-small cell lung cancer
OCSCC: Oral cavity squamous cell carcinoma
ORR: Overall response rate
OS: Overall survival
PBMC: Peripheral blood mononuclear cell
pCR: Complete pathological response
PDAC: Pancreatic adenocarcinoma
PD-1: Programmed death-1
PD-L1: Programmed death ligand-1
PFS: Progression-free survival
RFS: Recurrence-free survival
SCLC: Small-cell lung cancer
TCR: T cell receptor
TGF: Transforming growth factor
TIL: Tumur-infiltrating lymphocyte
TLR: Toll-like receptor
TMB: Tumor mutational burden
TME: Tumor microenvironment
TNBC: Triple-negative breast cancer
T-VEC: Talimogene laherparepvec
